# Variations in *HBA* gene contribute to high-altitude hypoxia adaptation via affected O_2_ transfer in Tibetan sheep

**DOI:** 10.1186/s12983-024-00551-1

**Published:** 2024-11-22

**Authors:** Pengfei Zhao, Xiong Ma, Jianming Ren, Lan Zhang, Yunxin Min, Chunyang Li, Yaoyao Lu, Ying Ma, Mingjie Hou, Hui Jia

**Affiliations:** Faculty of Chemistry and Life Sciences, Gansu Minzu Normal University, Hezuo, China

**Keywords:** Tibetan sheep, *HBA*, Hypoxia adaptation

## Abstract

Tibetan sheep are indigenous to the Qinghai-Xizang Plateau. Owing to the harsh hypoxic environment in this plateau, the hemoglobin (Hb) protein in Tibetan sheep has undergone adaptive changes over time. Hb is primarily responsible for transporting O_2_ and CO_2_ between the lungs and other tissues of the body. The α subunit of Hb, encoded by the *HBA* gene, is a crucial component of the protein. However, whether variations in the *HBA* gene sequence affect the adaptation of Tibetan sheep to high-altitude hypoxia remains unclear. In this study, we sequenced the *HBA* gene and identified three single nucleotide polymorphisms (SNPs). These SNPs were genotyped in Tibetan and Hu sheep using Kompetitive Allele-Specific PCR (KASP). The results showed that the frequencies of the AT genotype and H1H2 haplotype were higher in Tibetan sheep than in Hu sheep. Individuals with the AT genotype exhibited higher P_50_ levels, whereas those with the H1H2 haplotype exhibited lower PO_2_ and SaO_2_ levels. The higher P_50_ levels indicated that O_2_ was more readily released from oxygenated Hb into the tissues, with the lower PO_2_ and SaO_2_ levels facilitating this process. These findings indicate that variations in the *HBA* gene sequence contribute to enhancing O_2_ transfer efficiency in Tibetan sheep.

## Introduction

Adaptation is one of the fundamental characteristics of life activities. The Qinghai-Xizang Plateau, with an average altitude exceeding 4,000 m, is the world’s highest plateau. At this altitude, the available oxygen (O_2_) is < 60% of that at sea level [1]. Humans and animals inhabiting the Qinghai-Xizang Plateau have adapted well to the hypobaric hypoxic environment in the plateau. Tibetan sheep are indigenous to this plateau, distributed at an altitude of 2500–5000 m. At present, Tibetan sheep are the most widely raised livestock in the Qinghai-Xizang Plateau, becoming an important economic source for local farmers and herders. In addition, these sheep play an important role in social and cultural traditions [2] and in maintaining the stability of the alpine grassland ecosystem [3].

**Fig. 1:**
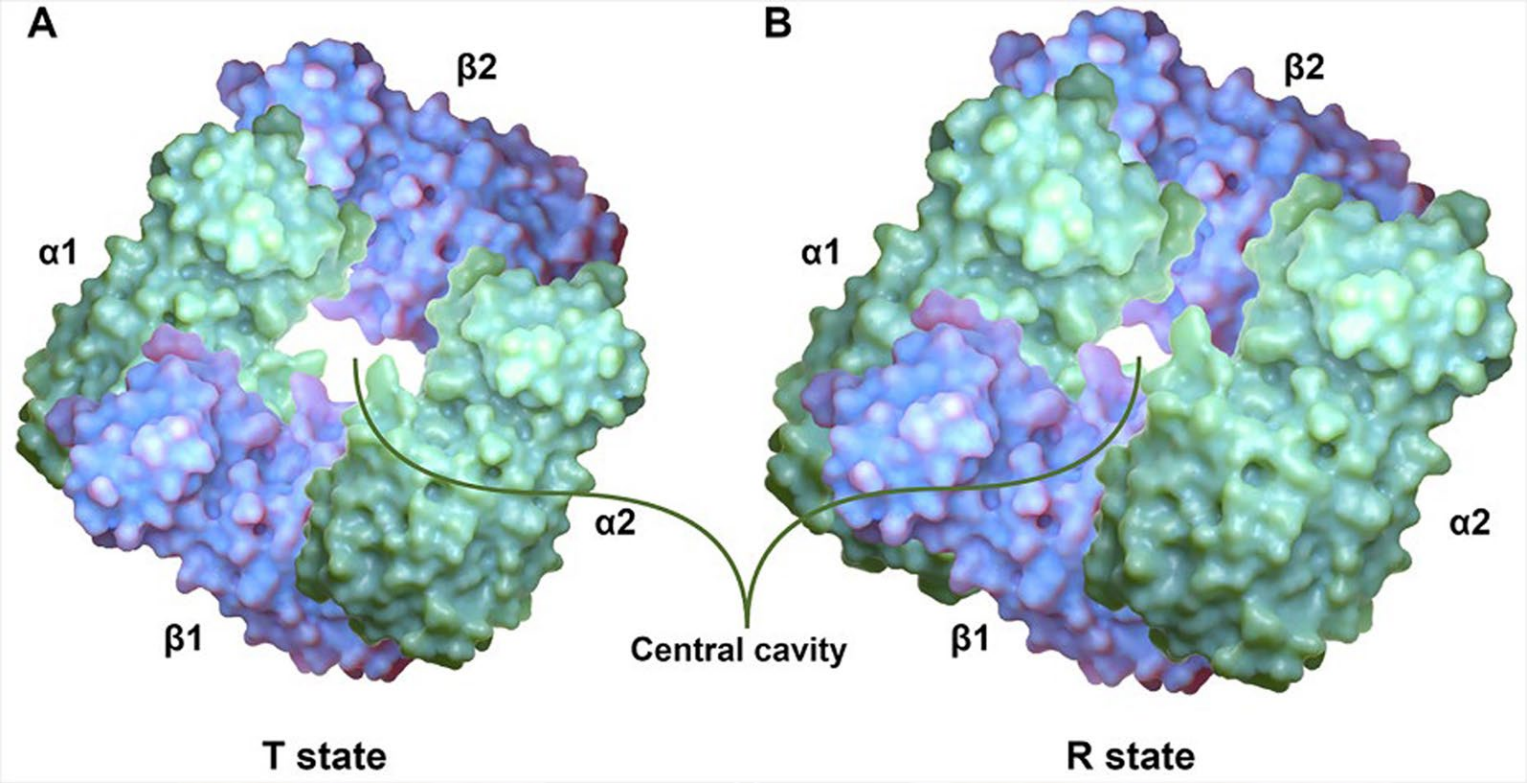
Structures of Hb deoxygenated (T state) **(A)** and oxygenated (R state) **(B)** formed by two α-chains (green) and two β-chains (blue). Note the larger central water cavity in the T state

Owing to severe environmental challenges caused by hypoxia in the Qinghai-Xizang Plateau, Tibetan sheep have undergone adaptive changes at the physiological, biochemical, and genetic levels. Therefore, they represent an ideal animal model for investigating the mechanisms underlying their adaptation to high-altitude hypoxia. Among the adaptive changes in response to high-altitude hypoxia, sequence variations in genes encoding hemoglobin (Hb) are particularly important. Hb is a tetramer consisting of two α and two β polypeptide chains of similar structure and dimension, and its main function is to transport O_2_ and CO_2_ between the lungs and other tissues of the body and maintain the acid–base balance of the blood [4]. The realization of this function depends on two states of Hb: tense (T) and relaxed (R). Two αβ dimers (α1β1 and α2β2) are aligned around an axis of symmetry, allowing Hb to form a central cavity that is wider in the T state and narrower in the R state, with the latter having a higher affinity for O_2_ [5] (Fig. 1). When a ligand (e.g., O_2_) binds to a subunit of Hb, it triggers a change in its tertiary conformation, which in turn triggers tertiary conformational changes in the other subunits. These changes lead to an increase in the affinity of the ligand for the other subunits, resulting in synergism between the subunits and eventually causing a structural change from the T to the R state [6]. These synergistic effects are observed only in tetramers consisting of two α and two β subunits. When these subunits are separated, the α and β subunits form an α dimer and a β tetramer, respectively; however, neither of them shows synergism [7]. Therefore, the heterogeneous tetrameric structure of Hb provides the structural basis for its binding to O_2_ in the lungs and the subsequent release of O_2_ in other tissues of the body.

The α subunit encoded by the *HBA* gene is an essential component of Hb. Studies have shown that mutations in the *HBA* gene are associated with adaptation to hypoxia. For instance, in plateau deer mice, variations in Hb subunit-encoding genes result in an increased Hb–O_2_ affinity and the inhibition of sensitivity to allosteric cofactors, such as chloride ions and 2,3-diphosphoglycerate [8], which contributes to the binding of Hb to O_2_. This phenomenon has also been observed in marmotine ground squirrels (subfamily Xerinae, tribe Marmotini) [9]. Moreover, some positively selected sites in the *HBA* gene have been associated with hypoxia tolerance in animals, such as cetaceans [10, 11]. The formation of a complex between Hb and glutathione can increase the affinity of Hb for O_2_ in humans and some other mammals [12, 13]. However, loss-of-function or loss-of-allele mutations in the *HBA* gene can lead to α-thalassemia, an autosomal recessive disease associated with the formation of β-tetramers and hemolytic anemia [14]. In addition to contributing to gas transport in erythrocytes, *HBA* is expressed in the vascular wall, especially at the junction of vascular endothelial cells and smooth muscle cells, and plays an important role in regulating nitric oxide (NO) signaling between these cells [15]. Although the *HBA* gene plays an essential role in gas transport and vascular function, the effects of sequence variations in this gene on the adaptation of Tibetan sheep to high-altitude hypoxia remain unclear. In this study, we investigated these effects using Tibetan sheep residing at altitudes of 3000 m and 4700 m and Hu sheep residing at an altitude of 100 m as animal models. Initially, we compared the levels of blood gas indicators between the two sheep breeds. Subsequently, we searched for variations in all exons and introns of the *HBA* gene via Sanger sequencing and genotyped the identified variants in both sheep breeds using Kompetitive Allele-Specific PCR (KASP). Finally, correlation analysis was performed between blood gas indicators and genotypes or haplotype combinations. Based on the data of blood gas indicators and *HBA* sequence variations, this study suggests that sequence variations in *HBA* partially explain how Tibetan sheep adapt to high-altitude hypoxia.

## Materials and methods

All animal experiments were conducted according to the animal protection and use guidelines established by the Ministry of Science and Technology of the People’s Republic of China (Approval number: 2006–398).

### Animal models and measurement of blood gas indicators

**Fig. 2:**
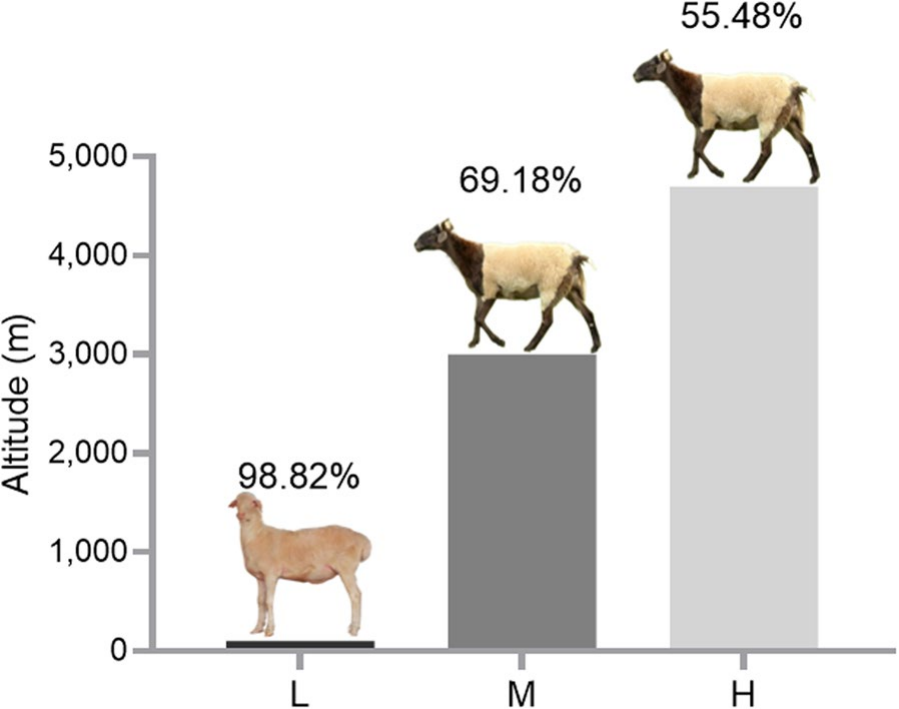
The Altitude distribution of sheep in this study: low-altitude (L) = 100 m; middle-altitude (M) = 3000 m; and high-altitude (H) = 4700 m. The percentage in this figure is the PO_2_ at that altitude relative to the sea level

Variations in all axons and introns of the *HBA* gene were examined in 341 Hu sheep residing at an altitude of 100 m (low altitude [L]; Hangzhou City, Zhejiang Province, China), 341 Tibetan sheep residing at an altitude of 3000 m (middle altitude [M]; Gannan Tibetan Autonomous Prefecture, Gansu Province, China), and 50 Tibetan sheep residing at an altitude of 4700 m (high altitude [H]; Naqu City, Tibet Autonomous Region, China) (Fig. 2). All sheep were naturally grazed with no supplemental feeding and were approximately 3 years old.

Jugular vein blood was collected from all sheep in 5-mL sodium heparin tubes. The blood was added dropwise onto a TFN paper (Munktell Filter AB, Falun, Sweden) to extract and purify DNA using a two-step procedure described previously [16]. Furthermore, jugular vein blood samples of 230, 282, and 42 sheep from the L, M, and H groups, respectively, were used for the assessment of blood gas indicators on an i-STAT blood gas analyzer (Abbott, Chicago, IL, USA). The blood gas indicators assessed included *pondus hydrogenii* (pH), partial pressure of oxygen (PO_2_), oxygen saturation (SaO_2_), partial pressure of carbon dioxide (PCO_2_), total carbon dioxide (TCO_2_), hematocrit (Hct), Hb concentration, glucose (Glu) concentration, base excess (BE), bicarbonate ion (HCO_3_^−^), serum sodium (Na^+^), serum potassium (K^+^), and serum calcium (Ca^2+^). The partial pressure of oxygen at which Hb was 50% saturated with O_2_ (P_50_) was calculated as an indicator of Hb–O_2_ affinity based on the pH, PO_2_, and SaO_2_ values using the following formula [17]:

$${{\varvec{P}}}_{50}{\varvec{s}}{\varvec{t}}{\varvec{d}}={\varvec{a}}{\varvec{n}}{\varvec{t}}{\varvec{i}}{\varvec{log}}\frac{{\varvec{l}}{\varvec{o}}{\varvec{g}}(\frac{1}{{\varvec{k}}})}{{\varvec{n}}};\boldsymbol{ }{\varvec{w}}{\varvec{h}}{\varvec{e}}{\varvec{r}}{\varvec{e}}\boldsymbol{ }\frac{1}{{\varvec{k}}}=\left[{\varvec{a}}{\varvec{n}}{\varvec{t}}{\varvec{i}}{\varvec{log}}({\varvec{n}}{\varvec{log}}{\varvec{P}}{{\varvec{O}}}_{2(7.4)})\right]\bullet \frac{100-{\varvec{S}}{\varvec{a}}{{\varvec{O}}}_{2}}{{\varvec{S}}{\varvec{a}}{{\varvec{O}}}_{2}}$$

The Hill constant (n) for Hb was set to 2.7. The PO_2_ in venous blood at 37℃ was converted to PO_2_ at pH 7.4 using the following formula:

$${\varvec{log}}{\varvec{P}}{{\varvec{O}}}_{2\left(7.4\right)}={\varvec{log}}{\varvec{P}}{{\varvec{O}}}_{2}-[0.5\left(7.40-{\varvec{p}}{\varvec{H}}\right)]$$

### PCR and genotyping

The *HBA* gene has a total of three exons and two introns. The primers for *HBA* were designed using the Primer 5.0 tool (Table 1). The genomic DNA of 20 Tibetan sheep from the M group was used to amplify the sequence of *HBA*, followed by the sequencing of all amplicons. Primer synthesis, amplification, and sequencing were performed by Sangon Biotech Co., Ltd. (Shanghai, China). The resulting sequences were analyzed using BLAST to detect single nucleotide polymorphisms (SNPs), and KASP was subsequently used for genotyping. Both BLAST and KASP were performed by Gentides Biotech Co., Ltd. (Wuhan, China) (Table 2). After genotyping, the fluorescence data were analyzed using an enzyme marker with fluorescence resonance energy transfer function, and genotyping maps were generated using the LGC-OMEGA software.

**Table 1:** Primer information for the *HBA* gene

Gene	Forward primer sequence (5' – 3')	Forward primer sequence (5' – 3')
*HBA*	GACCCCGACACCCTACACGCTC	GGGGAACTTGGTTCAGCAGATTCTG

Due to the short sequence of the *HBA* gene (≈ 769 bp), a pair of primers can amplify all its exons and introns

**Table 2:** The genotype primers of three SNPs of *HBA* gene

Gene	Position	Genotype primer sequence (5' to 3')
*HBA*	g.758709 A > T	F (A): GAAGGTGACCAAGTTCATGCTCGGCAACGCTGGAGCTTA
		F (T): GAAGGTCGGAGTCAACGGATTCGGCAACGCTGGAGCTTT
		R: CGGTGCTCACCTCTCCAGAG
	g.758789 G > T	F (G): GAAGGTGACCAAGTTCATGCTGCGCGTCCTTGTCCCG
		F (T): GAAGGTCGGAGTCAACGGATTGGCGCGTCCTTGTCCCT
		R: GGTGAAGAGGCGGGAAAGC
	g.758808 T > C	F (T): GAAGGTGACCAAGTTCATGCTCCGCTCGGCCTGAGCCT
		F (C): GAAGGTCGGAGTCAACGGATTCGCTCGGCCTGAGCCC
		R: GGGGAAGTAGGTCTTGGTGGTG

### Statistical analysis

The SPSS (version 19.0) software was used to compare blood gas indicators between Tibetan and Hu sheep using the following general linear model: Y = µ + A + S + A*S + ε, wherein Y represents the phenotypic observation; µ represents the mean population; A and S represent the effects of altitude and sex, respectively; A*S represents the reciprocal effect of altitude and sex; and ε represents random error. The blood gas indicators of sheep in the L group were used as baseline, and changes in the blood gas indicators of sheep in the M and H groups were compared with those in the L group (*P* < 0.05).

After the successful genotyping of SNPs in the *HBA* gene, allele frequencies, genotype frequencies, effective number of alleles (Ne), heterozygosity (He), homozygosity (Ho), and polymorphism information content (PIC) were calculated using formulas reported by Botstein et al. [18]. Hardy–Weinberg equilibrium (HWE) was tested using the chi-square (χ^2^) test. Linkage disequilibrium analysis and haplotype construction were performed using Haploview (version 4.2) [19]. The correlation between blood gas indicators and different genotypes or haplotype combinations was analyzed in the SPSS (version 19.0) software using the following general linear model: Y = µ + G + A + S + G*A + A*S + G*S + G*A*S + ε, wherein Y represents the phenotypic observation; μ represents the mean population; G represents the effect of the genotype (when analyzing haplotype combinations, G is replaced with H, that is, the effect of the haplotype combination); A and S represent the effects of altitude and sex, respectively; G*A, A*S, and G*S represent the reciprocal effects of genotype and altitude, altitude and sex, and genotype and sex, respectively; G*A*S represents the reciprocal effects of genotype, altitude, and sex; and ε represents random error. All experimental data were expressed as the mean ± SD. Differences were estimated using Duncan's test, with a *P* value of < 0.05 or < 0.01 indicating significant or extremely significant differences.

## Results

### Differences in blood gas indicators between Hu and Tibetan sheep

**Fig. 3:**
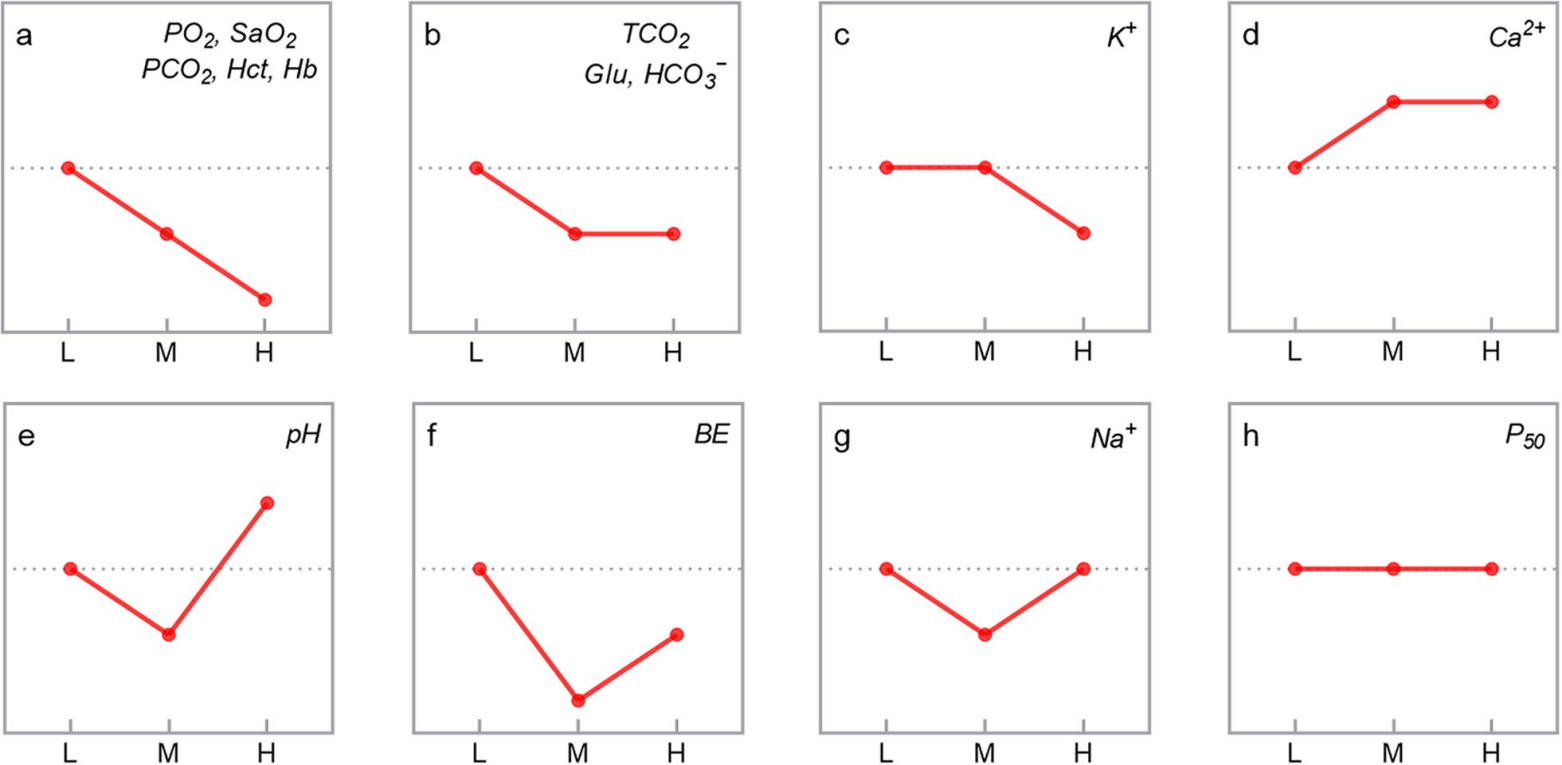
Changing trends of blood-gas indicators with increasing altitude in sheep, a diagonal line between two points means that the difference is significant, the blood-gas indicators in the patterns have corresponding changing trends

The levels of blood gas indicators were compared among the L, M, and H groups of sheep. The results showed that the levels of PO_2_, SaO_2_, PCO_2_, Hct, Hb, TCO_2_, Glu, HCO_3_^−^, and K^+^ decreased with an increase in the altitude (*P* < 0.05), whereas Ca^2+^ levels showed the opposite trend (*P* < 0.05). The pH, BE, and Na^+^ levels decreased initially but increased subsequently with an increase in the altitude (*P* < 0.05). On the contrary, P_50_ was not significantly different among the three groups (*P* > 0.05) (Table 3 and Fig. 3).

**Table 3:** Blood-gas indicators of sheep at different altitudes

Blood-gas indicators	Altitudes		
	L	M	H
PO_2_ (mmHg)	42.67 ± 7.68^a^	34.59 ± 6.44^b^	24.05 ± 3.43^c^
SaO_2_ (%)	76.98 ± 8.33^a^	64.33 ± 9.90^b^	46.40 ± 10.57^c^
P_50_ (mmHg)	26.57 ± 0.48	26.57 ± 0.39	26.66 ± 0.38
PCO_2_ (mmHg)	42.54 ± 6.52^a^	38.18 ± 6.06^b^	32.93 ± 4.65^c^
TCO_2_ (mmol/L)	27.64 ± 2.21^a^	23.74 ± 2.96^b^	24.10 ± 2.86^b^
Hct (%)	36.10 ± 5.83^a^	34.38 ± 2.97^b^	28.07 ± 3.50^c^
Hb (g/dL)	12.27 ± 1.99^a^	11.69 ± 1.01^b^	9.55 ± 1.20^c^
Glu (mg/dL)	67.55 ± 7.43^a^	62.09 ± 12.00^b^	62.81 ± 7.47^b^
pH	7.41 ± 0.06^b^	7.38 ± 0.06^c^	7.45 ± 0.07^a^
BE (mmol/L)	1.93 ± 2.46^a^	− 2.43 ± 3.24^c^	− 0.83 ± 3.20^b^
HCO_3_^−^ (mmol/L)	26.64 ± 2.22^a^	22.57 ± 2.82^b^	23.12 ± 2.75^b^
Na^+^ (mmol/L)	144.95 ± 2.23^a^	143.50 ± 2.41^b^	144.33 ± 1.22^a^
K^+^ (mmol/L)	5.09 ± 0.72^a^	5.07 ± 1.24^a^	4.34 ± 0.45^b^
Ca^2+^ (mmol/L)	1.26 ± 0.08^b^	1.30 ± 0.09^a^	1.31 ± 0.07^a^

Differences in blood-gas indicators of sheep at low (L), middle (M) and high (H) altitudes. Partial pressure of oxygen (PO_2_), Oxygen saturation (SaO_2_), Half-saturation oxygen partial pressure (P_50_), Partial pressure of carbon dioxide (PCO_2_), Total carbon dioxide (TCO_2_), Hematocrit (Hct), Hemoglobin concentration (Hb), Glucose concentration (Glu), Pondus Hydrogenii (pH), Base excess (BE), Bicarbonate ion (HCO_3_^−^), Serum sodium (Na^+^), Serum potassium (K^+^) and Serum calcium (Ca^2+^) are shown. Different lowercase letters in the same line indicate that the difference was significant (*P* < 0.05)

### Variations in the *HBA* gene in Hu and Tibetan sheep

**Fig. 4:**
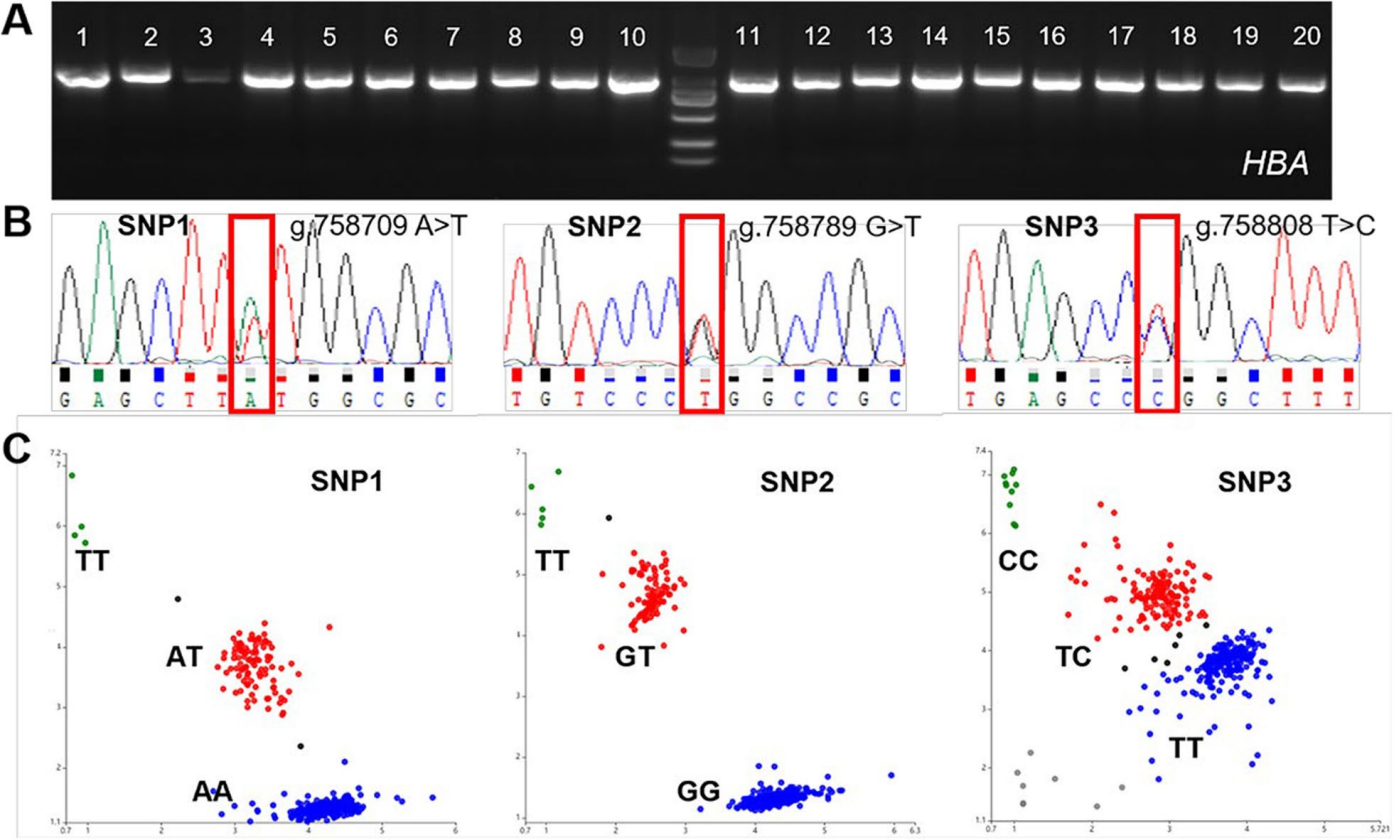
PCR amplicons agarose gel electrophoresis (**A**) and Sanger-sequencing (**B**) results (the overlapping peak indicates the SNPs), and KASP genotyping assay results of three SNPs of the *HBA* gene (**C**)

The PCR amplicons of *HBA* were analyzed via agarose gel electrophoresis (a 2% gel), and the results showed that the bands of the amplicons were clear, complete, and free of contamination (Fig. 4A). Sanger sequencing of the amplicons showed that exon 1 of *HBA* was mutated at g.758709 (A > T), whereas intron 1 was mutated at g.758789 (G > T) and g.758808 (T > C). These mutations were named SNP1, SNP2, and SNP3, respectively (Fig. 4B).

The three SNPs were genotyped using KASP in all sheep. The results revealed three genotypes for SNP1, SNP2, and SNP3 each (Fig. 4C). The dominant genotypes of SNP1, SNP2, and SNP3 in the *HBA* gene were AA (0.961), GG (0.953), and TT (0.821) in the L group; AA (0.745), GG (0.747), and TT (0.607) in the M group; and AA (0.604), GG (0.540), and TT = TC (0.476) in the H group, respectively. In addition, the dominant alleles were A, G, and T; A, G, and T; and A, G, and T, respectively (Table 4). The nucleotide transversion from A to T in SNP1 led to a tyrosine-to-phenylalanine amino acid change; however, SNP2 and SNP3, which were located in the first intron of *HBA*, did not result in any amino acid change.

**Table 4:** Genotype frequency and allele frequency of three SNPs of *HBA* gene

Positions	Genotype	Genotype frequency			Allele	Allele frequency		
		L (n)	M (n)	H (n)		L	M	H
SNP1 A > T	AA	0.961 (324)	0.745 (254)	0.604 (29)	A	0.981	0.864	0.792
	AT	0.039 (13)	0.237 (81)	0.375 (18)	T	0.019	0.136	0.208
	TT	0.000 (0)	0.018 (6)	0.021 (1)				
SNP2 G > T	GG	0.953 (321)	0.747 (254)	0.540 (27)	G	0.976	0.865	0.750
	GT	0.047 (16)	0.235 (80)	0.420 (21)	T	0.024	0.135	0.250
	TT	0.000 (0)	0.018 (6)	0.040 (2)				
SNP3 T > C	TT	0.821 (151)	0.607 (205)	0.476 (20)	T	0.908	0.787	0.714
	TC	0.174 (32)	0.361 (122)	0.476 (20)	C	0.092	0.213	0.286
	CC	0.005 (1)	0.033 (11)	0.048 (2)				

### Population genetic analysis of the three SNPs of the *HBA* gene

Population genetic analysis revealed that all three SNPs were less polymorphic (PIC < 0.25) and moderately polymorphic (0.25 < PIC < 0.5) in the L and H groups, respectively. SNP1 and SNP2 were less polymorphic and SNP3 was moderately polymorphic in the M group. All three SNPs had higher Ho than He in all three groups and conformed to HWE (*P* > 0.05). In addition, SNP3 and SNP1 had the highest and lowest Ne in the three groups, respectively (Table 5).

**Table 5:** Population genetics analysis of three SNPs of *HBA* gene

Positions	Altitudes	PIC^1^	He^2^	Ho^3^	Ne^4^	HWE^5^
SNP1 A > T	L	0.0371	0.0378	0.9622	1.0393	*P* > 0.05
	M	0.2078	0.2355	0.7645	1.3081	*P* > 0.05
	H	0.2755	0.3299	0.6701	1.4922	*P* > 0.05
SNP2 G > T	L	0.0453	0.0464	0.9536	1.0486	*P* > 0.05
	M	0.2066	0.2340	0.7660	1.3054	*P* > 0.05
	H	0.3047	0.3750	0.6250	1.6000	*P* > 0.05
SNP3 T > C	L	0.1536	0.1677	0.8323	1.2051	*P* > 0.05
	M	0.2791	0.3353	0.6647	1.5044	*P* > 0.05
	H	0.3249	0.4082	0.5918	1.6897	*P* > 0.05

^1^ Polymorphism information content; ^2^ heterozygosity; ^3^ homozygosity; ^4^ effective allele numbers; ^5^ Hardy–Weinberg equilibrium

**Fig. 5:**
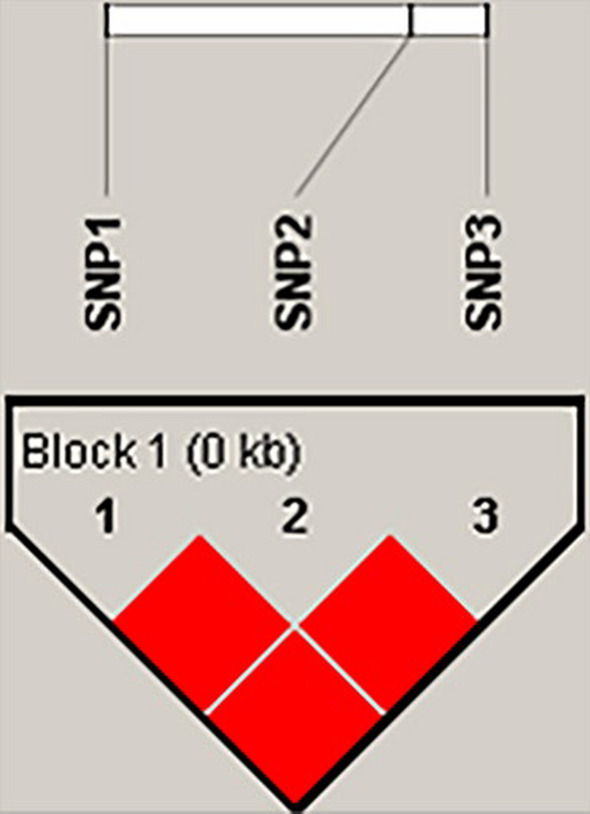
Linkage disequilibrium analysis of three SNPs of *HBA* gene

Linkage disequilibrium analysis showed that the three SNPs of the *HBA* gene exhibited strong linkage (D’ > 0.9) (Fig. 5). Haplotype analysis revealed three haplotypes in the L and M groups and four haplotypes in the H group. After these haplotypes were combined, three haplotype combinations with frequencies greater than 0.03 were identified in each group (Table 6).

**Table 6:** Haplotypes and haplotype combinations of three SNPs of *HBA* gene

Haplotype	Positions			Frequency			Haplotype combination	Frequency		
	SNP1	SNP2	SNP3	L	M	H		L	M	H
H1 (AGT)	A	G	T	0.907	0.787	0.729	H1H1	0.833	0.602	0.488
H2 (TTC)	T	T	C	0.022	0.136	0.213	H1H2	0.033	0.226	0.366
H3 (AGC)	A	G	C	0.071	0.077	0.036	H1H3	0.133	0.142	0.098
H4 (ATC)	A	T	C	——	——	0.021				

### Correlation of the genotypes and haplotype combinations with blood gas indicators

Correlation analysis between SNP genotypes and blood gas indicators showed that individuals with the TT genotype at SNP1 had lower PO_2_, SaO_2_, PCO_2_, TCO_2_, and BE than individuals with the AA genotype (*P* < 0.05) and lower SaO_2_ and P_50_ than individuals with the AT genotype (*P* < 0.05). Individuals with the TT genotype at SNP2 had lower PO_2_ and SaO_2_ than individuals with the GG and GT genotypes (*P* < 0.05), lower P_50_ than individuals with the GT genotype (*P* < 0.05), and lower TCO_2_ and BE than individuals with the GG genotype (*P* < 0.05). Individuals with the CC genotype at SNP3 had lower PO_2_, SaO_2_, and PCO_2_ (*P* < 0.05) but higher pH (*P* < 0.05) than individuals with the TT and TC genotypes (Table 7).

**Table 7:** The effects of different genotypes of *HBA* gene three SNPs on blood-gas indicators

Positions	Blood-gas indicators	Genotype		
		AA	AT	TT
SNP1 A > T	PO_2_ (mmHg)	37.97 ± 8.76^a^	33.27 ± 6.61^ab^	27.67 ± 10.12^b^
	SaO_2_ (%)	69.40 ± 12.55^a^	62.63 ± 12.14^a^	52.00 ± 21.00^b^
	P_50_ (mmHg)	26.58 ± 0.44^ab^	26.67 ± 0.50^a^	26.20 ± 0.09^b^
	PCO_2_ (mmHg)	40.01 ± 6.87^a^	37.78 ± 6.13^ab^	32.40 ± 5.23^b^
	TCO_2_ (mmol/L)	25.20 ± 3.34^a^	24.29 ± 3.10^ab^	22.33 ± 2.52^b^
	Hct (%)	34.99 ± 5.27	33.67 ± 4.32	32.00 ± 6.08
	Hb (g/dL)	11.90 ± 1.80	11.45 ± 1.48	10.87 ± 2.06
	Glu (mg/dL)	53.21 ± 25.40	60.93 ± 13.40	68.00 ± 19.08
	pH	7.40 ± 0.06	7.40 ± 0.06	7.42 ± 0.09
	BE (mmol/L)	− 0.28 ± 3.59^a^	− 1.38 ± 3.56^ab^	− 3.33 ± 3.79^b^
	HCO_3_^−^ (mmol/L)	25.10 ± 10.59	23.40 ± 3.03	21.07 ± 2.57
	Na^+^ (mmol/L)	143.84 ± 6.61	143.95 ± 2.36	145.00 ± 1.00
	K^+^ (mmol/L)	5.05 ± 1.01	4.97 ± 1.24	4.65 ± 0.35
	Ca^2+^ (mmol/L)	1.31 ± 0.31	1.30 ± 0.09	1.33 ± 0.10
		GG	GT	TT
SNP2 G > T	PO_2_ (mmHg)	37.99 ± 8.73^a^	33.52 ± 7.29^a^	26.75 ± 8.46^b^
	SaO_2_ (%)	69.39 ± 12.55^a^	63.07 ± 12.43^a^	48.75 ± 18.34^b^
	P_50_ (mmHg)	26.57 ± 0.44^ab^	26.67 ± 0.50^a^	26.25 ± 0.13^b^
	PCO_2_ (mmHg)	40.02 ± 6.84	37.65 ± 6.29	34.88 ± 6.54
	TCO_2_ (mmol/L)	25.19 ± 3.33^a^	24.49 ± 3.15^ab^	22.75 ± 2.22^b^
	Hct (%)	35.02 ± 5.24	33.37 ± 4.41	32.25 ± 4.99
	Hb (g/dL)	11.91 ± 1.78	11.34 ± 1.50	10.95 ± 1.69
	Glu (mg/dL)	53.44 ± 25.28	61.30 ± 13.45	66.50 ± 15.86
	pH	7.40 ± 0.06	7.41 ± 0.07	7.40 ± 0.09
	BE (mmol/L)	− 0.32 ± 3.57^a^	− 1.09 ± 3.66^ab^	− 3.25 ± 3.10^b^
	HCO_3_^−^ (mmol/L)	25.07 ± 10.63	23.59 ± 3.07	21.48 ± 2.25
	Na^+^ (mmol/L)	143.84 ± 6.64	144.04 ± 2.38	145.00 ± 0.82
	K^+^ (mmol/L)	5.05 ± 1.01	4.93 ± 1.20	4.60 ± 0.26
	Ca^2+^ (mmol/L)	1.31 ± 0.31	1.30 ± 0.09	1.32 ± 0.08
		TT	TC	CC
SNP3 T > C	PO_2_ (mmHg)	37.38 ± 9.27^a^	34.20 ± 6.63^a^	28.83 ± 7.55^b^
	SaO_2_ (%)	68.19 ± 13.24^a^	64.00 ± 11.77^a^	56.00 ± 16.70^b^
	P_50_ (mmHg)	26.56 ± 0.44	26.64 ± 0.47	26.49 ± 0.42
	PCO_2_ (mmHg)	39.07 ± 6.46^a^	38.81 ± 6.94^a^	33.10 ± 4.28^b^
	TCO_2_ (mmol/L)	24.67 ± 3.27	24.64 ± 3.23	23.50 ± 3.56
	Hct (%)	34.59 ± 4.72	33.85 ± 4.28	31.67 ± 5.72
	Hb (g/dL)	11.76 ± 1.61	11.51 ± 1.46	10.77 ± 1.95
	Glu (mg/dL)	55.90 ± 23.40	59.99 ± 14.94	63.33 ± 14.07
	pH	7.40 ± 0.06^b^	7.40 ± 0.07^b^	7.44 ± 0.08^a^
	BE (mmol/L)	− 0.86 ± 3.62	− 1.17 ± 3.56	− 1.67 ± 4.50
	HCO_3_^−^ (mmol/L)	24.84 ± 13.08	23.69 ± 3.11	22.48 ± 3.67
	Na^+^ (mmol/L)	143.57 ± 8.02	143.87 ± 2.75	144.83 ± 2.14
	K^+^ (mmol/L)	5.01 ± 1.05	5.03 ± 1.23	4.63 ± 0.22
	Ca^2+^ (mmol/L)	1.31 ± 0.28	1.33 ± 0.36	1.31 ± 0.08

Different lowercase letters in the same line indicate that the difference was significant (*P* < 0.05)

Correlation analysis between haplotype combinations and blood gas indicators showed that individuals with the H1H2 haplotype had lower PO_2_ and SaO_2_ than individuals with the H1H1 and H1H3 haplotypes (*P* < 0.05) and lower PCO_2_ than individuals with the H1H3 haplotype (*P* < 0.05) (Table 8).

**Table 8:** The effects of different haplotype combinations of *HBA* gene three SNPs on blood-gas indicators

Blood-gas indicators	Haplotype combination		
	H1H1	H1H2	H1H3
PO_2_ (mmHg)	37.48 ± 9.27^a^	33.16 ± 6.34^b^	35.90 ± 6.45^a^
SaO_2_ (%)	68.37 ± 13.21^a^	62.28 ± 12.20^b^	66.45 ± 10.82^a^
P_50_ (mmHg)	26.56 ± 0.44	26.69 ± 0.52	26.57 ± 0.39
PCO_2_ (mmHg)	39.06 ± 6.50^ab^	37.86 ± 6.08^b^	40.45 ± 7.64^a^
TCO_2_ (mmol/L)	24.67 ± 3.28	24.24 ± 2.99	24.98 ± 3.50
Hct (%)	34.62 ± 4.74	33.81 ± 4.32	34.42 ± 3.96
Hb (g/dL)	11.77 ± 1.62	11.49 ± 1.48	11.71 ± 1.36
Glu (mg/dL)	55.91 ± 23.60	61.69 ± 12.27	57.08 ± 18.05
pH	7.40 ± 0.06	7.40 ± 0.06	7.39 ± 0.06
BE (mmol/L)	− 0.85 ± 3.62	− 1.52 ± 3.45	− 1.04 ± 3.55
HCO_3_^−^ (mmol/L)	24.86 ± 13.19	23.29 ± 2.91	24.01 ± 3.31
Na^+^ (mmol/L)	143.58 ± 8.08	143.92 ± 2.41	144.63 ± 3.17
K^+^ (mmol/L)	5.00 ± 1.04	4.97 ± 1.28	5.18 ± 1.19
Ca^2+^ (mmol/L)	1.31 ± 0.28	1.31 ± 0.09	1.37 ± 0.58

Different lowercase letters in the same line indicate that the difference was significant (*P* < 0.05)

## Discussion

Blood gas indicators play a crucial role in the adaptation of animals to high-altitude hypoxic environments. PO_2_ accounts for 21% of the barometric pressure in the Qinghai-Xizang Plateau. The barometric pressure decreases with altitude, leading to hypobaric hypoxia in animals inhabiting the plateau [1]. Consistently, in this study, the PO_2_ in the blood of sheep decreased with an increase in altitude (*P* < 0.05). This decrease in PO_2_ may be the reason for the simultaneous decrease in SaO_2_ (*P* < 0.05) [20]. Furthermore, an increase in Hb concentration is considered a typical adaptive response to high altitudes. However, in this study, Hb concentration and Hct levels decreased with an increase in altitude (*P* < 0.05). Similar phenomena have been observed in Tibetans [21, 22] and Tibetan horses [23]. These findings may be attributed to the weak response of some physiological and biochemical indicators to hypoxia in species indigenous to high-altitude regions [24]. Moderate increases in Hb concentration and Hct levels indicate an increase in the number of O_2_ carriers; however, excessive increases in Hb concentration and Hct levels can increase the viscosity of the blood and result in increased resistance, which may cause pulmonary hypertension and damage microcirculation. Therefore, animals inhabiting high-altitude regions may enhance their O_2_ transport efficiency through other means, such as by increasing the respiratory rate [25, 26], heart rate [27], and plasma volume [28]. A higher respiratory rate indicates higher pulmonary ventilation, i.e., more CO_2_ is exhaled, resulting in a decrease in PCO_2_, TCO_2_, and HCO_3_^−^, accompanied by an increase in pH and BE. This hypoxia-induced shift of the acid–base balance toward alkalinity reduces sympathetic nervous tension and cardiovascular constriction [29], favoring the avoidance of pulmonary hypertension. In addition, a lower PO_2_ and Hb concentration indicate inhibition of O_2_ delivery; however, animals inhabiting high-altitude environments tend to be more efficient in their use of O_2_. For example, more use of carbohydrates as substrates for energy metabolism, that is, increased oxidation of carbohydrates and glycolysis, leads to a decrease in blood Glu levels [30]. This phenomenon is observed in both Tibetans and Sherpas [31, 32], as carbohydrates provide more ATP when the same amount of O_2_ is consumed [33]. Altogether, the changes in blood gas indicators in response to an increase in altitude help Tibetan sheep overcome chronic hypobaric hypoxia.

The protein encoded by the *HBA* gene is one of the subunits that comprise Hb, which plays an important role in maintaining O_2_ homeostasis and acid–base balance in the body [4]. This study showed that the frequency of the AT genotype at SNP1 of the *HBA* gene was higher in Tibetan sheep residing at medium (M, 3000 m) and high (H, 4700 m) altitudes than in Hu sheep residing at low (L, 100 m) altitude and that individuals with the AT genotype had higher P_50_ (*P* < 0.05). When P_50_ was elevated, the O_2_ dissociation curve shifted to the right, resulting in a lower Hb–O_2_ affinity, which indicated that O_2_ was more readily dissociated from oxygenated Hb to mitigate tissue hypoxia. A high Hb–O_2_ affinity does not enhance the organism’s ability to adapt to the hypoxic environment [34]; on the contrary, the organism may adapt to the hypoxic environment by decreasing Hb–O_2_ affinity, that is, by increasing P_50_. The change in the nucleotide from A to T in SNP1 results in an amino acid change from tyrosine to phenylalanine. This non-synonymous mutation may affect the structure and function of the Hb protein, resulting in an increase in P_50_. This change may explain the higher frequency of the AT genotype in Tibetan sheep residing at M and H altitudes. However, this finding requires further validation in vitro and in vivo. The polymorphic information content of SNP1 was higher at both M and H altitudes than at L altitude, and is in Hardy–Weinberg equilibrium, indicates some potential for selection. SNP2 and SNP3 were found to be located in the first intron of the *HBA* gene and did not cause any amino acid changes. However, the correlation analysis found that the genotypes of the two SNPs were correlated with some blood gas indicators. Therefore, we speculate that the two SNPs affect blood gas indicators by influencing variable shearing of the *HBA* gene [35, 36], or by participating in the regulation of transcriptional activity of the *HBA* gene [37]. And again, the above speculations requires further validation in vitro and in vivo.

When investigating the effects of genetic variations on phenotype, the results obtained by analyzing the relationship between an SNP and the phenotype are limited, and richer and more reliable information can be obtained by deriving haplotypes from multiple SNPs and analyzing the effects of these haplotypes on the phenotype [38, 39]. In this study, Linkage disequilibrium analysis revealed that all three SNPs of the *HBA* gene had strong linkage, which satisfies the requirement for constructing haplotypes [39]. Based on this, three haplotype combinations with frequencies higher than 0.03 were constructed. The frequency of the H1H2 haplotype was higher in Tibetan sheep at M and H altitudes than in Hu sheep at L altitude, and individuals with the H1H2 haplotype had lower PO_2_, SaO_2_, and PCO_2_ values (*P* < 0.05). Lower PO_2_ and SaO_2_ allow O_2_ to dissociate from Hb more readily [40], this contributes to the alleviation of tissue hypoxia. In general, animals adapt to hypoxia in two main ways: firstly, increased efficiency of O_2_ delivery; and secondly, increased efficiency of O_2_ use. Oxygen dissociates more readily from Hb reflecting the increased efficiency of O_2_ delivery, and animals inhabiting high-altitudes regions have enhanced carbohydrates metabolism, especially glycolysis, reflecting the increased efficiency of O_2_ use. Since more energy is released from carbohydrate oxidation than from fatty acid or amino acid oxidation per mole of oxygen consumed [33]. This is a common adaptive response to hypoxia [31, 32, 41]. The lower PCO_2_ value in individuals with the H1H2 haplotype may be attributed to enhanced glycolysis, as glycolysis occurring in the cytoplasm does not produce CO_2_ [42], resulting in lower PCO_2_.

## Conclusion

This study reveals that the g.758709, g.758789, and g.758808 variants in the *HBA* gene are associated with an increased O_2_ transfer efficiency in Tibetan sheep. The frequencies of the AT genotype and H1H2 haplotype were higher in Tibetan sheep than in Hu sheep. Individuals with the AT genotype and H1H2 haplotype exhibited higher P_50_ levels and lower PO_2_ and SaO_2_ levels. The higher P_50_ level facilitates the release of O_2_ from oxygenated Hb into the tissues, and the lower PO_2_ and SaO_2_ levels enhance this process. Altogether, these factors promote O_2_ transfer efficiency and enable Tibetan sheep to adapt to hypoxia in the Qinghai-Xizang Plateau.

## Data Availability

The datasets during and/or analyzed during the current study available from the corresponding author on reasonable request.
